# Adsorption-Site- and Orientation-Dependent Magnetism of a Molecular Switch on Pb(100)

**DOI:** 10.1021/acsnano.4c17183

**Published:** 2025-02-14

**Authors:** Arnab Banerjee, Niklas Ide, Yan Lu, Richard Berndt, Alexander Weismann

**Affiliations:** † Institut für Experimentelle und Angewandte Physik, Christian-Albrechts-Universität, 24098 Kiel, Germany; ‡ Department of Physics, Nanchang University, Nanchang 330031, People’s Republic of China

**Keywords:** adsorption geometry, molecular magnetism, phthalocyanines, scanning tunneling microscopy, Yu−Shiba−Rusinov states, superconductors

## Abstract

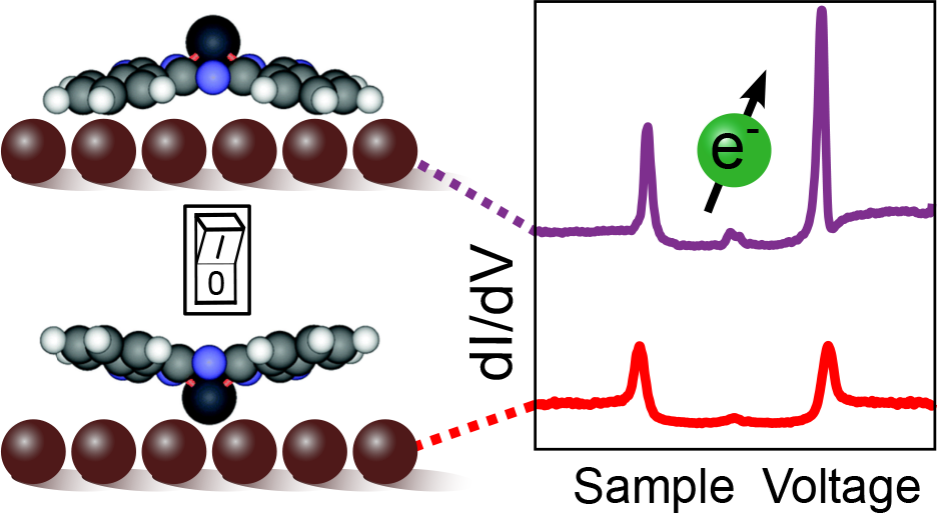

Tin phthalocyanine (SnPc) has been studied on superconducting Pb(100) using scanning tunneling microscopy and spectroscopy. Isolated molecules adsorb with their Sn ion below (SnPc↓) or above (SnPc↑) the molecular plane. These geometries lead to different adsorption sites, molecular orientations, and energies of the frontier orbitals. A transition from SnPc↑ to SnPc↓ can be induced by extracting electrons from a single molecule. Density functional theory (DFT) calculations reproduce the observed geometries and indicate that a positive charge of the molecules facilitates the ↑–↓ transition. The molecular orientations are essentially determined by the σ-orbitals on the peripheral N atoms and exhibit minimum distances of their lone pairs from the nearest Pb substrate atoms. This binding scheme, which implies a direct relationship between the adsorption site and the molecular orientation, is consistent with many previous observations on other substrates. In molecular islands, single molecules can be forced onto less favorable adsorption sites. This leads to a strong Yu–Shiba–Rusinov state of SnPc↓ at top sites revealing an induced molecular spin. Similarly, the spin observed from SnPc↑ on hollow sites is quenched by their conversion to SnPc↓. The calculated lowest unoccupied molecular orbital energies are consistent with these spin-state transitions.

## Introduction

Magnetic molecules hold great promise as building blocks for future nanotechnology applications.^1−3^ Among them, molecules that undergo a geometric change coupled with spin switching are of particular interest.^4^ Various mechanisms behind this phenomenon have been studied extensively, including spin crossover,^5^ coordination-induced spin switching,^6,7^ electron-transfer coupled spin transition,^8^ and valence tautomerism.^9^ These mechanisms typically involve transition metal ions within molecular complexes.

In this study, we demonstrate spin switching in a phthalocyanine (Pc) molecule that is diamagnetic in the gas phase and notably lacks a transition metal ion. Phthalocyanines can adopt either a planar or shuttlecock geometry depending on the size of the central atom. For instance, cations such as Pb, In, and Sn are too large to fit into the Pc-macrocycle, leading to a shuttlecock-shaped geometry. Upon adsorption onto a planar surface, these complexes can adopt two distinct conformations, with the metal ion pointing either toward the vacuum ↑ or toward the substrate ↓ as revealed by scanning tunneling microscope (STM) imaging.

By injecting current into SnPc molecules, transitions between the SnPc↑ and SnPc↓ geometries have been demonstrated on Ag(111) surfaces.^10,11^ The binding and the geometric transition on this surface have also been studied with DFT calculations.^12,13^ The electronic structure and spin state of adsorbed molecules are highly influenced by structural parameters, as well as interactions with the substrate and neighboring molecules.^14−22^ In particular, electrostatic properties like the quadrupole moment, induced dipole moments, and the role of image charges play important roles.^23,24^ Consequently, the geometric transition of nonplanar phthalocyanines offers a promising avenue for controlling electronic and magnetic states. Notably, we recently observed such an effect in InPc.^25^

Here, we investigate SnPc on Pb(100) using STM combined with DFT calculations. Similar to the behavior observed on Ag(111), a geometrical transition can be induced using the STM. Taking advantage of the superconducting substrate, we find that the magnetic state is modified by the transition between the two conformers. Remarkably, the molecular spin can be switched on or off depending on the molecular environment. This provides new insights into the binding and orientation of the SnPc molecule on the surface and characterizes conditions, under which SnPc becomes paramagnetic.

## Results and Discussion

### Island Formation

**Figure 1:**
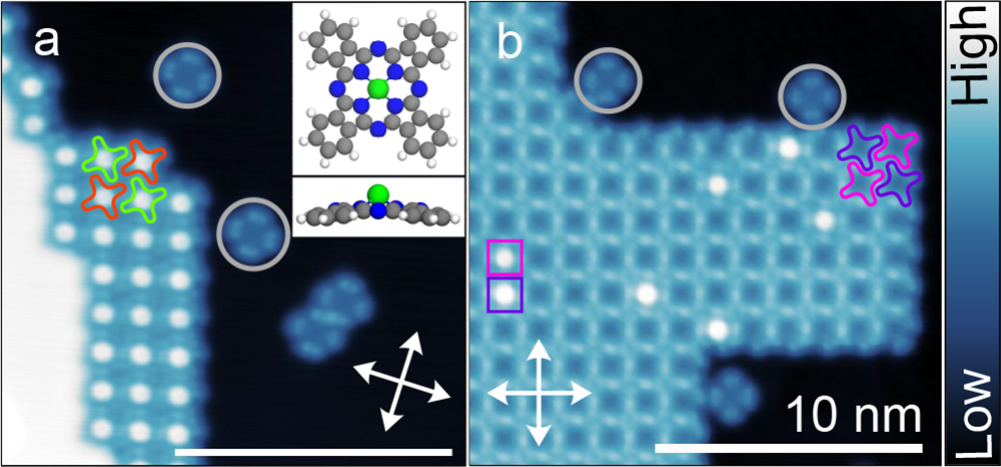
Topographs of a submonolayer amount of SnPc on Pb(100). (a) Island containing only SnPc↑ molecules along with some substrate area (black) and an upper terrace (white) (*V* = 20 mV). The molecules form a checkerboard pattern. Substrate ⟨110⟩ directions are indicated by white arrows. Red and green molecular outlines indicate the conformations α_1_ and α_2_, which differ by the orientation of their lobes with respect to the substrate, namely, 45 and 50° respectively. (b) STM topograph (*V* = 50 mV, *I* = 60 pA) of an island containing predominantly SnPc↓ molecules. Violet and pink outlines show the conformations β_1_ and β_2_ with orientations of 28 and 20°, respectively. Squares indicate the examples of SnPc↑ molecules that substitute ↓ molecules on β_1_ and β_2_ sites. Gray circles indicate the examples of isolated H_0_Pc molecules on the terraces. The color scale covers ranges between 290 and 250 pm in panels (a) and (b), respectively.

Figure 1 shows two constant-current topographs of a Pb(100) surface covered with a submonolayer amount of SnPc. The molecules are arranged in two types of islands that predominantly contain either SnPc↑ (Figure 1a) or SnPc↓ (Figure 1b) molecules. The ↑ and ↓ orientations of SnPc are readily distinguished from the apparent height at the center of each molecules (high or low). Similar to previous observations for PbPc,^24^ a few isolated molecules with their lobes oriented parallel to ⟨110⟩ substrate directions are also found (examples indicated with gray circles). These molecules lost their Sn ion and are denoted H_0_Pc. Closer inspection of the data reveals checkerboard-like superstructures with two molecules per unit cells. The square unit cells of SnPc lattice are rotated by 60 and 45° with respect to substrate lattice for ↑ and ↓ islands, respectively. The ↑ island exhibits a superstructure that was previously observed from H_2_Pc and PbPc,^24^ namely . The ↓ island, however, has a more dense  superstructure previously observed from AlPc.^18^ Interestingly, the former structure is accompanied by facetting of the Pb steps, which adopt the orientation of the molecular rows. A similar effect was previously observed from a porphyrin derivative and CuPc.^21,26^

The superstructure of Figure 1a is comprised of a checkerboard pattern with two nonequivalent molecular orientations, denoted as α_1_ and α_2_. The molecular isoindole lobes are oriented at angles of 45 and 50° with respect to ⟨110⟩ directions of the substrate. This is identical to the superlattice of A-islands reported for PbPc^24^ and H_2_Pc^27^ on Pb(100). In contrast, the molecules in Figure 1b are rotated by 28 and 20° and designated as β_1_ and β_2_, respectively. Occasionally, SnPc↑ molecules are embedded in SnPc↓ islands. ↑ molecules that substitute a β_1_ ↓ molecule appear slightly higher (height difference 25 pm) compared to those on β_2_ positions. Examples are indicated by squares in Figure 1b. The orientations of the molecules and the lattice vectors of the superstructure are identical to islands of AlPc on same substrate.^18^ Various characteristics of the conformers may be found in Table 1.

**Table 1:** Structural Parameters and YSR Energies^a^

	Sn position	position in unit cell	angle (°)	site	*E*_YSR_ (meV)
Isolated	↑		0	t	–
	↑		45	t	–
	↓		±25	h	–
Island	↑	α_1_	45	t	1.12
	↑	α_2_	50	t	–
	↑	β_1_	28	h	1.04
	↑	β_2_	20	h	–
	↓	α_1_^*^	45^†^	t	0.14
	↓	α_2_^*^	50^†^	t	0.0
	↓	β_1_^*^	28^†^	h	–
	↓	β_2_^*^	20^†^	h	–

a Hollow and top adsorption sites are abbreviated as h and t. ^†^ indicates the presumed value because of confinement in an island. ^*^ indicates the preparation by induced ↑ to ↓ transition. – indicates the absence of YSR state.

Using the STM tip, SnPc molecules can be moved from island edges onto clean substrate areas (see Methods section). In contrast, manipulation attempts for H_0_Pc molecules were not successful, indicating a stronger coupling to the substrate.

### Adsorption Geometries of Isolated Molecules

**Figure 2:**
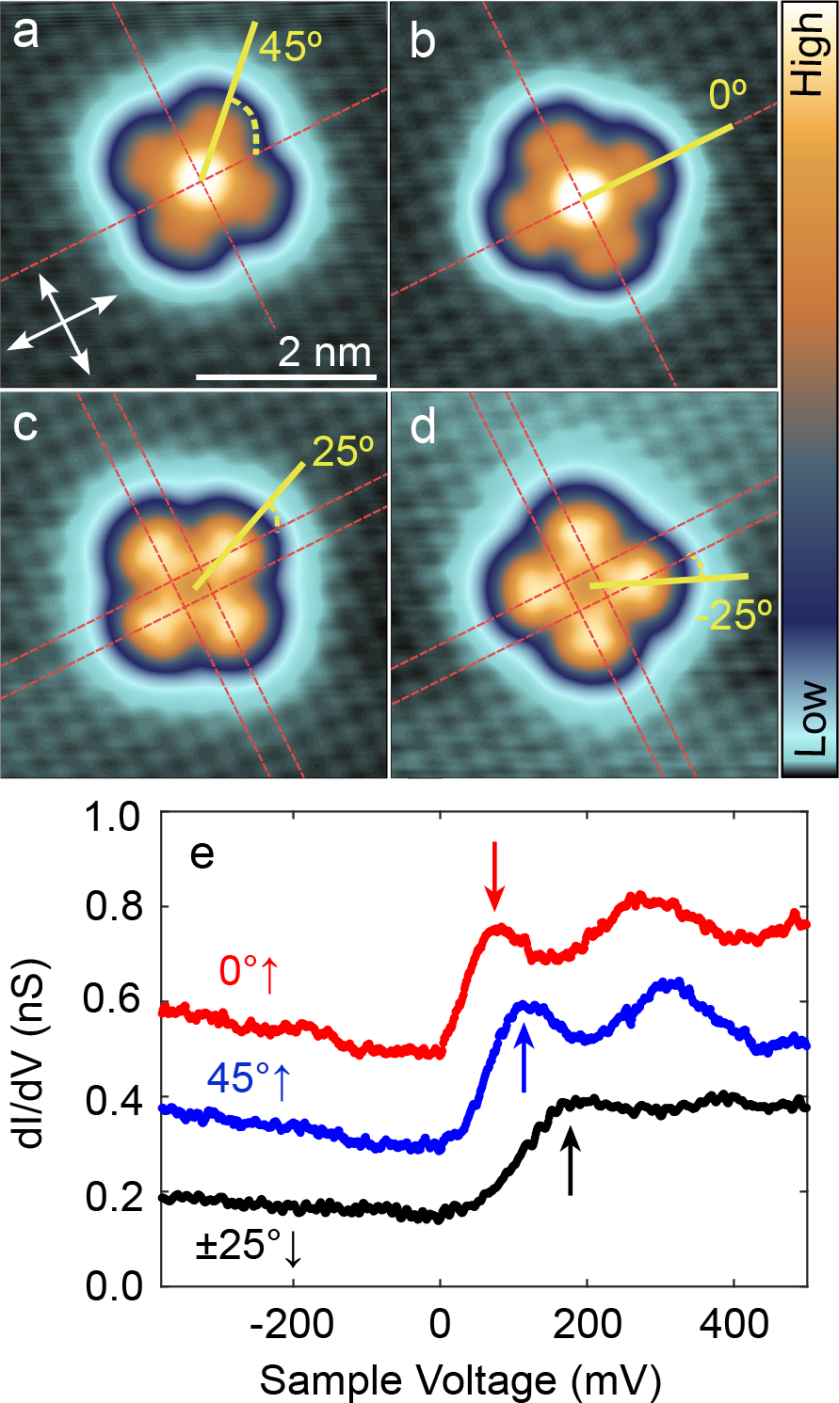
Adsorption geometry and electronic structure of isolated molecules. (a–d) Constant-current topographs of isolated SnPc complexes recorded at low sample voltages (10–20 meV) and elevated currents (600–800 pA). The Pb(100) substrate lattice is resolved around the molecules, which enables a determination of the adsorption sites and the azimuthal orientations. Dashed red lines and white arrows indicate ⟨110⟩ directions of the Pb lattice. The orientations of the molecules are defined by the angle of their lobes with respect to these substrate directions. The SnPc↑ molecules in panels (a) and (b) exhibit a central protrusion, are centered atop Pb atoms, and are oriented at 0 and 45°. The SnPc↓ molecules in panels (c) and (d) show minimum at their center, are located above 4-fold hollow sites, and enclose angles of ±25° with the substrate directions. The color range spans 290 and 200 pm in (a, b) and (c, d), respectively. (e) d*I*/d*V* spectra recorded above the center of each molecule. The approximate positions of the lowest-energy peaks, which are attributed to the lowest unoccupied molecular orbital, are marked with arrows. Parameters used prior to define the tip height for spectroscopy: *V* = 600 mV and *I* = 200 pA. All spectra are vertically shifted by 0.2 nS for clarity.

Figure 2 displays atomically resolved STM topographs of isolated SnPc molecules. The isolated molecules were prepared by laterally moving them onto the terrace from there original sites in molecular islands or at step edges (see Supporting Figure 1). The center of SnPc↑ molecules, which exhibit a central protrusion in the images, is adsorbed to top sites of the Pb mesh as evident from an extrapolation of the Pb lattice observed nearby (Figure 2a,b, red dashed lines). We find angles of 0 and 45° between the isoindole lobes and the ⟨110⟩ directions of the substrate. In contrast, SnPc↓ molecules occupy hollow sites and are orientated at angles of ±25° (Figure 2c,d).

The different adsorption geometries come along with differences in the electronic structure of the molecules. Figure 2e shows d*I*/d*V* spectra measured above the center of SnPc↑ and SnPc↓ molecules with different orientations. All spectra display a pair of maxima with ≈200 mV separation, which we attribute to the lowest unoccupied molecular orbital (LUMO) and a related vibronic excitation. A clear dependency on the adsorption geometry is also observed. The LUMO (arrows in Figure 2e) is centered around 80, 110, and 180 mV for SnPc↑ with 0 and 45° orientations and for SnPc↓ molecules, respectively.

### DFT Calculations

**Figure 3:**
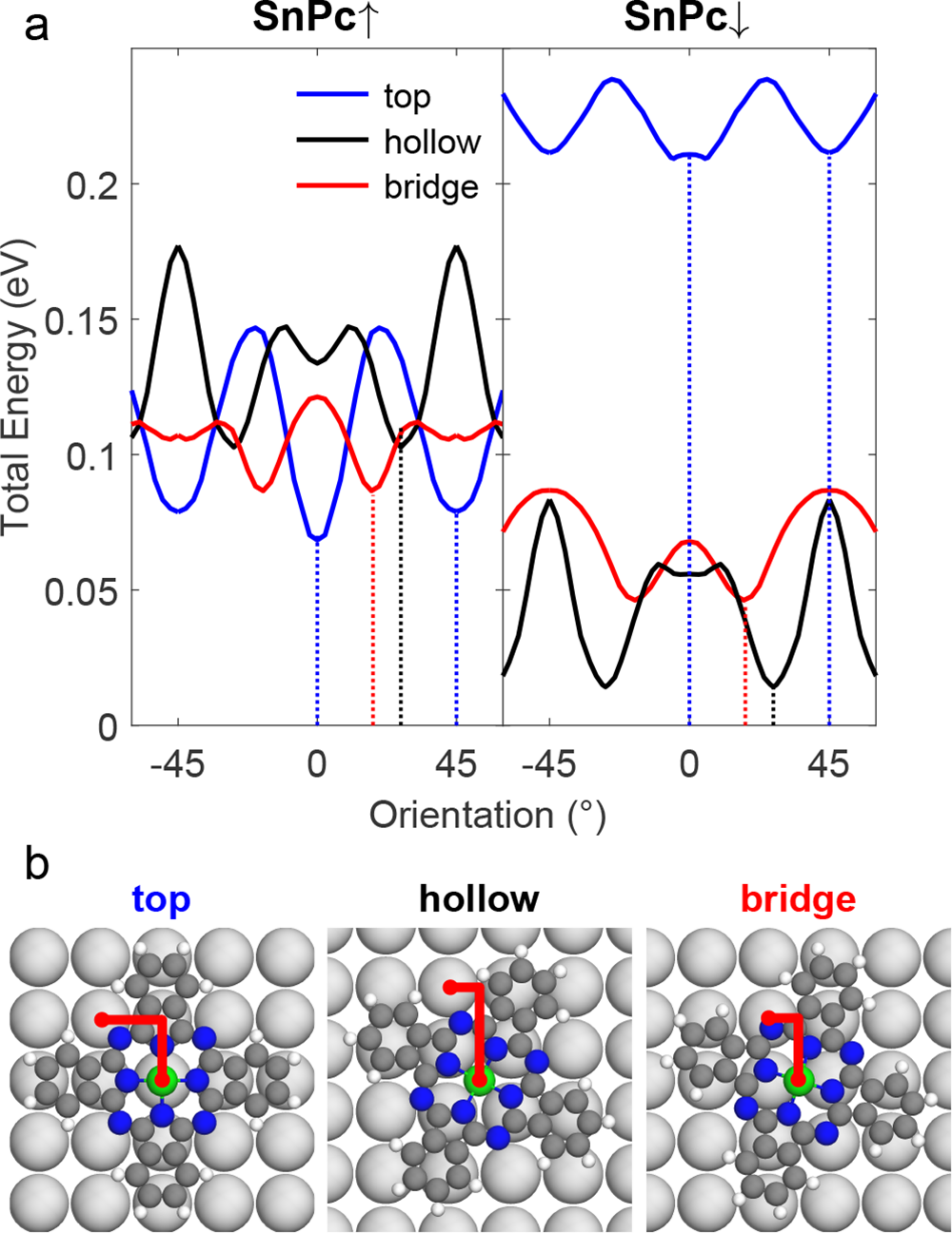
Calculated structures. (a) DFT total energy vs molecular orientation of SnPc↑ (left) and SnPc↓ (right) for top (blue), bridge (red), and hollow (black) adsorption sites. Red (black) vertical dotted lines in (a) mark angles of 18° (27°), where energy minima are located for bridge (hollow) sites. Blue dotted lines mark the minima for top-site adsorption at 0 and 45°. (b) Molecular models with γ = 0° (top), γ = 18° (bridge), and γ = 27° (hollow). Red lines in panel (b) mark the displacement between the center of the molecule and Pb substrate atoms in proximity of the aza-nitrogen atoms.

We used DFT to determine the optimized adsorption sites and orientations of isolated SnPc↑ and SnPc↓ molecules on the Pb(100) surface. Figure 3a shows the calculated total energies *E*(γ) of SnPc↑ and SnPc↓ as a function of the molecular orientation γ, defined as the angle between an isoindole lobe and a substrate ⟨110⟩ direction. The angular energy profiles for top (blue), hollow (black), and bridge (red) adsorption sites exhibit similar qualitative trends for both conformers, with local minima occurring at the same orientations. For bridge sites, the preferred orientation is γ = ±18°, while for hollow sites, it is ±27°, as indicated by the red and black dotted vertical lines. When the molecule is centered on a top site (blue), the energy profile shows two local minima at 0 and 45° (blue dotted lines). Although the local minima occur at the same orientations for both conformers, the absolute energies differ: for SnPc↓, the top site is approximately 200 meV higher in energy compared to the hollow site, whereas for SnPc↑, the top site is energetically favored. These DFT results align well with experimentally observed adsorption sites and orientations within the experimental uncertainty.

Figure 3b illustrates the minimum energy configurations with γ = 0, 27, and 18° centered on top, hollow and bridge sites, respectively. The components (marked in red) of the vector connecting the Sn atom to the nearest Pb atom beneath the bridging-aza nitrogen of the SnPc macrocycle help explaining the preferred orientations. Assuming that this distance is minimized, the adsorption angles are expected to be 45° – arctan(0.5/1.5) = 26.6° for hollow sites and 45° – arctan(0.5/1.0) = 18.4° for bridge sites, consistent with our findings.

**Figure 4:**
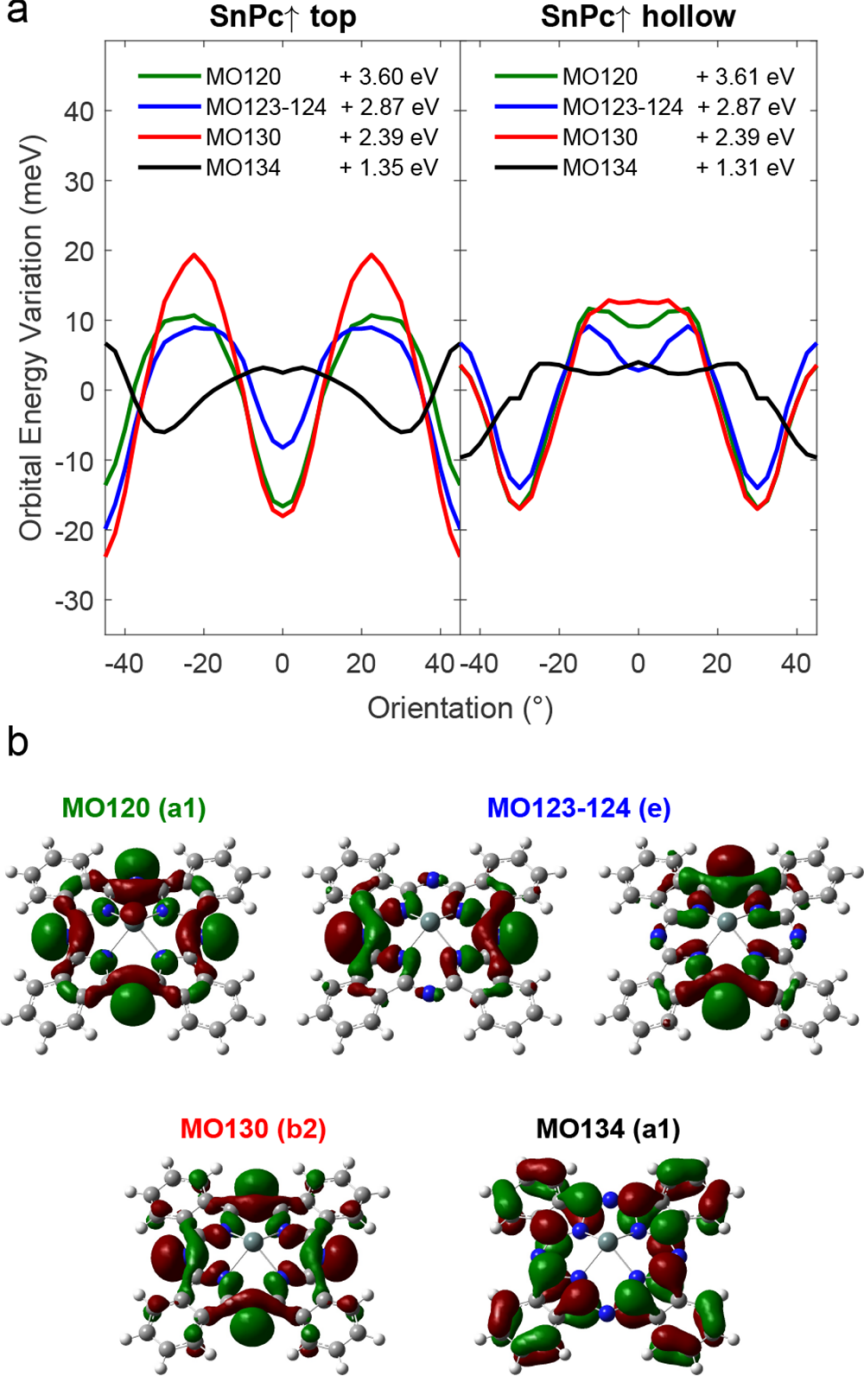
(a) Energy variation vs molecular orientation of those occupied molecular σ-orbitals showing the strongest angular dependence (red, green, blue) and a π-MO (black) for comparison. (b) Wave function isosurfaces of the molecular orbitals analyzed in panel (a). MO120, MO123–124, and MO130 contain the four lone electron pairs at the bridging aza-nitrogen atoms with pronounced (*p*_*x*_, *p*_*y*_) characters.

Next, we examined how the projected density of states (PDOS) evolves with the molecular orientation (Figure 4a). Since the total energy *E* is determined by the sum of the energies of all occupied states, we focused on identifying the molecular orbitals (MOs) most affected by changes in orientation. By projecting the molecular density of states onto specific atomic orbitals, we were able to attribute PDOS resonances to individual gas-phase MOs.

We found that the states with the strongest angular energy dependence are primarily associated with the atomic *p*_*x*_ and *p*_*y*_ orbitals of the aza-nitrogen atoms. The lone electron pairs on the four aza-nitrogen atoms are distributed across four doubly occupied molecular σ-orbitals: MO120, MO123–124 and MO130, which are indicated in Figure 4b. These orbitals exhibit a pronounced sensitivity to molecular orientation mirroring the angular dependence of *E*(γ). For comparison, the evolution of a molecular π-Orbital (MO134) is also shown, which shows a weaker and partially inverted angular dependence. This indicates that shifts in the molecular σ-orbitals arising from the coupling between the aza-nitrogen atoms and substrate Pb atoms play a crucial role in determining the variation of the total energy with the molecular orientation.

Our analysis revealed that the bridging-aza nitrogen atoms of the Pc macrocycle tend to minimize their distance to the nearest Pb atom of the substrate. Identical angles should be observed on other (001) surfaces This hypothesis is supported by previous findings. For example, AlPc on Pb(100) was observed to adsorb at bridge sites, fluctuating between γ = ±18°.^28^ Similarly, ClAlPc on Cu(100) adsorbs on hollow sites with γ = ±27°,^29^ while NiPc and CuPc on Ag(100) adopt orientations near ±30°.^30^ Thus, the binding mechanism identified here likely applies to other phthalocyanines on various (001) surfaces. Moreover, the observed molecular orientations could serve as indicators of adsorption sites, even in the absence of atomically resolved imaging.

### Geometric Transition of SnPc

**Figure 5:**
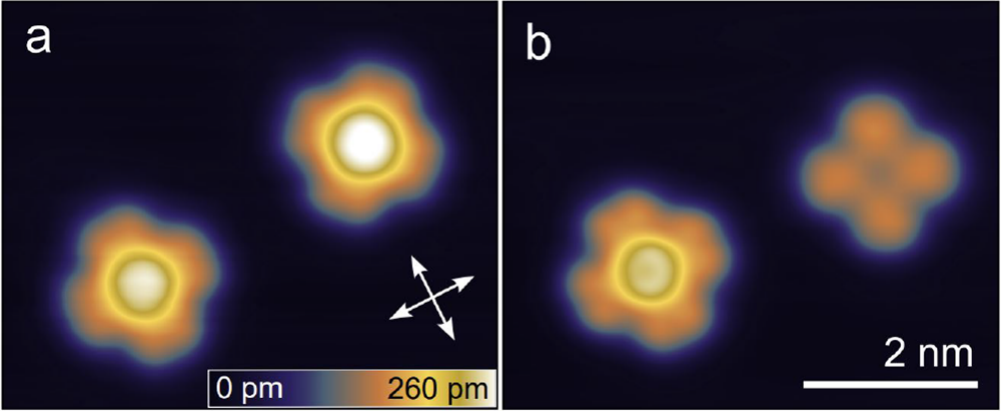
Current-induced switching. Topographs of two isolated SnPc complexes (a) before (*V* = 324 mV) and (b) after (*V* = 50 mV, *I* = 50 pA) manipulating the molecule on the right, as detailed in the text. While both molecules initially were in the ↑ state, the molecule on the right was converted to SnPc↓ by the manipulation procedure. In addition, its azimuthal orientation changed from 45 to 25°, as expected for the ↓ state.

The conversion of SnPc↑ to SnPc↓ is demonstrated in Figure 5. Two isolated SnPc↑ molecules were prepared by moving them away from a step edge. Next, the tip was placed above the center of the molecule on the right and the sample voltage *V*, was progressively lowered to more negative values beyond −1.6 V in constant current mode until an abrupt movement of the tip toward the molecule was observed. Finally, the area was imaged again using a nonperturbative voltage (Figure 5b). While the molecule on the left remained unchanged, the manipulated molecule has been converted to the ↓ state and its azimuthal orientation has changed to −25° the value typically observed from isolated SnPc↓ molecules. d*I*/d*V* spectra of the molecule recorded before and after the transition were virtually identical to the corresponding spectra shown in Figure 2e, which further confirms the attribution to SnPc↑ and SnPc↓. A rotation from 0° or 45 to ±25° along with a lateral translation from a top to hollow site was always observed.

The switching reproducibly occurred close to *V* = −1.6 V and did not depend significantly on the tunneling current. The deviation of this threshold from the highest vibrational mode energies of SnPc (≈400 meV) suggests that vibrational excitation alone is not a likely cause of the transition. On the other hand, the calculated energy of the highest occupied molecular orbital (HOMO) is close to the observed threshold. This suggests that electron removal from the HOMO triggers the transition from SnPc↑ to SnPc↓. The results of DFT calculations for neutral and positively charged SnPc shown in the Supporting Figure 2 of Wang et al.^10^ are in line with this proposal. From this, the positively charged molecule is more flat and the estimated energy barrier of the ↑–↓ transition is reduced by a factor of 2 to ≈1.5 eV.

### Spin Switching in Islands

Below we take advantage of the switchability of the complexes within islands to prepare SnPc↓ molecules in nonfavorable orientations and probe their spin state. Similarly, a minority of SnPc↑ molecules exists in SnPc↓ islands and their spin states are investigated.

#### SnPc↑ Islands

**Figure 6:**
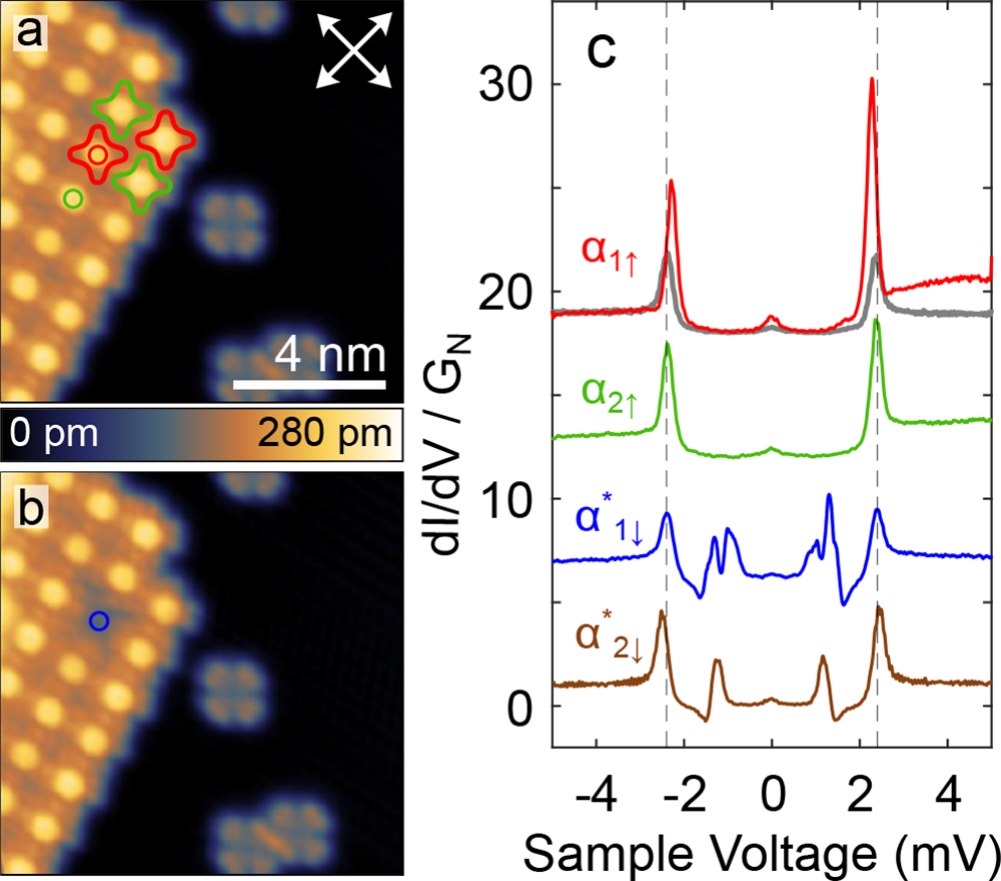
Spin-state switching in SnPc*↑* island. (a) Topograph (*V* = 20 mV) of a SnPc*↑* island and a substrate area with isolated H_0_Pc molecules. Some molecules with α_1_ and α_2_ conformations are indicated by red and green outlines. Substrate ⟨110⟩ directions are marked by white arrows. (b) Topograph (*V* = 50 mV) after switching an α_1_ SnPc↑ to SnPc↓, denoted  (blue circle). (c) d*I*/d*V* spectra of α_1↑_, α_2↑_, and  conformations acquired at the locations marked by colored circles in panels (a) and (b). The  spectrum was measured elsewhere. A gray line shows the spectrum of the coherence peaks of bare Pb(100) recorded with the same tip. Dashed lines indicate the corresponding voltages. All spectra are normalized to identical conductance at *V* < −4.8 mV and vertically offset for clarity. The tip position was stabilized at *V* = 5 mV before disabling the feedback.

Figure 6a displays an area from a SnPc↑ island. The molecule indicated by a green circle was manipulated using an elevated voltage and underwent a transition to SnPc↓ (Figure 6b, blue circle). Panel (c) shows a comparison of the d*I*/d*V* spectra of molecules (colors) and the Pb substrate (gray). Pristine α_1↑_ molecules (red curve) exhibit a clear peak height asymmetry that signals the presence of a YSR state with *E*_YSR_ = 1.12 meV. A smalller asymmetry also observed from the α_2↑_ conformation (green curve) is not due to such a state. This effect is rather caused by a slope of the background conductance. The d*I*/d*V* map recorded on such an island (see Supporting Figure 2) at a voltage slightly higher than the superconducting coherence peaks depicts a decrease in conductance over α_1↑_ type molecules, characteristic for the YSR state. The manipulated molecules  (blue) and  (brown), however, show distinct YSR features well inside the superconducting gap at energies *E*_YSR_ = 0.14 and 0 meV, respectively. In other words, the YSR state of SnPc↓ molecules that are forced α_1_ or α_2_ orientations in an SnPc↑ environment are drastically different from those of their SnPc↑ neighbors and those of their original orientations.

Bound states located near the center of the superconducting energy gap are highly unlikely to arise from resonant potential scattering by a nonmagnetic impurity, which induces the so-called Shibata states.^31^ For these states to approach midgap, the hybridization must be significantly smaller than the superconducting gap parameter Δ. This leads to the conclusion that  and  are paramagnetic.

#### SnPc↓ Islands

**Figure 7:**
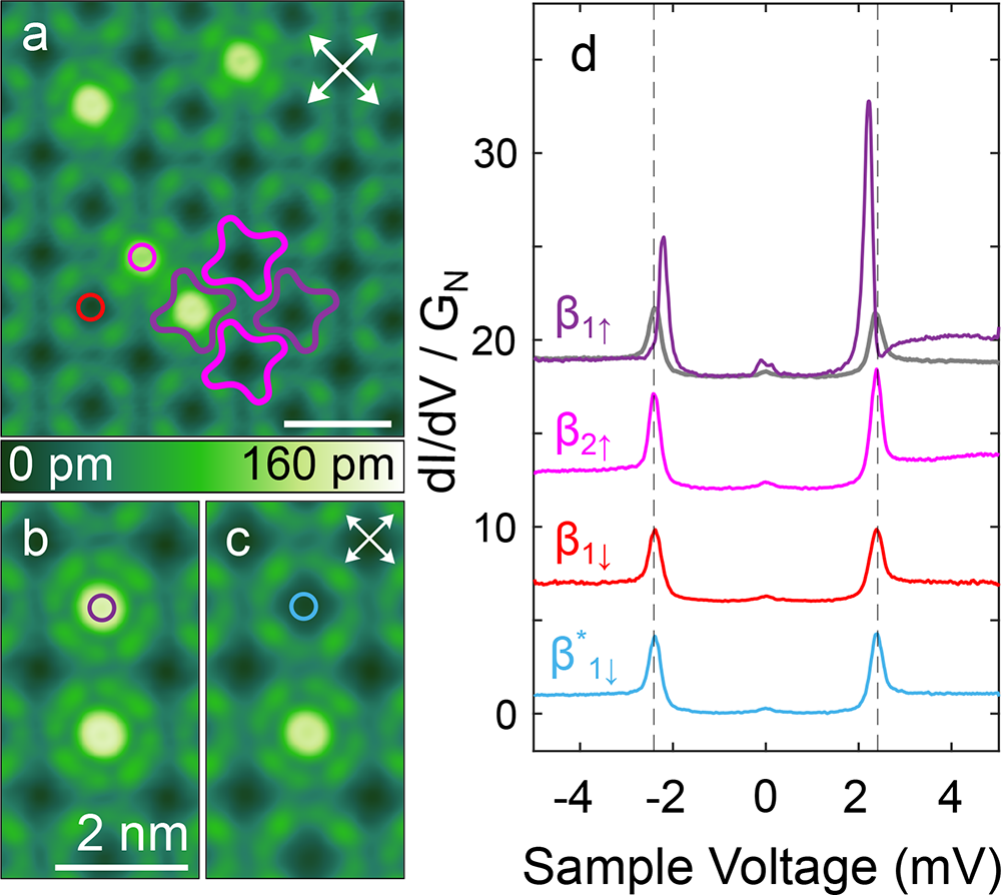
Spin-state switching in SnPc*↓* island. (a) Topograph (*V* = 10 mV) of a SnPc↓ island. Some molecules with β_1_ and β_2_ conformations are indicated by violet and pink outlines. Substrate ⟨110⟩ directions are marked by white arrows. (b) Topograph (*V* = 10 mV) before inducing a transition of the β_1_ SnPc↑ molecules indicated with a violet circle. (c) Topograph (*V* = 20 mV) recorded after the transition. (d) d*I*/d*V* spectra of β_1↑_, β_2↑_, β_1↓_, and  conformations acquired at the locations marked by colored circles in panels (a–c). A gray line shows the spectrum of the coherence peaks of bare Pb(100) recorded with the same tip. Dashed lines indicate the corresponding voltages. All spectra are normalized to identical conductance at *V* < −4.8 mV and vertically offset for clarity. The tip position was stabilized at *V* = 5 mV before disabling the feedback.

Figure 7a shows the checkerboard pattern of a self-assembled island that is mainly comprised of SnPc↓ molecules and a few SnPc↑ impurities. The current-induced transition of such a SnPc↑ complex is presented in panels (b) and (c). The typical protrusion of SnPc↑ (violet circle in b) is changed to the depression of SnPc↓ (blue circle in c). Spectroscopic data of the various conformers are shown in Figure 7d. A conductance map at *V* = 2.5 mV (see Supporting Figure 3) also depicts the presence of YSR state over β_1↑_ molecule. The manipulated conformer (, blue curve) does not show any indication of YSR states. There is no peak height asymmetry and the peak positions match those of the substrate spectrum (gray) very well. Its spectrum is identical to that of pristine β_1↓_ (red curve) as expected. Both cases are different from pristine β_1↑_ with a clear YSR state at *E*_YSR_ = ±1.04. As to β_2↑_, a minor asymmetry is discernible but it turned out to result from a linear LUMO-related background.

The presence and absence of YSR states of β_1↑_ and β_2↑_ molecules is reminiscent of the case of AlPc, which arranges itself in the same superstructure.^18^ The different orientations of the two molecules in the unit cell affect the electrostatic dipole fields of polarized C–H-bonds that lower the electrostatic potential on β_1_ sites relative to β_2_ and thus lead to pronounced YSR states.

To rationalize the measured YSR-energies, we analyzed the characteristics of the LUMO for different orientations and adsorption sites using DFT calculations. These calculations were performed for isolated molecules on Pb(100) and thus do not account for interaction with neighboring molecules, which lower the orbital energies. However, they do capture the degree of hybridization of the LUMO with the substrate and include the orbital shift caused by the interaction of the molecule with its image charges. From the DFT molecular density of states ϱ(*E*), we computed the quantities *E*_L_ = *N*∫ *E*ϱ(*E*)d*E* (the LUMO energy) and Γ_L_ = *N*∫ |*E* – *E*_L_|ϱ(*E*)d*E* (the LUMO width), where *N*^–1^ = ∫ ϱ(*E*)d*E*, integrating within an energy window of ±0.5 eV around the Fermi energy. Additionally, the LUMO occupation was estimated as *n*_L_ = *N*∫ ϱ(*E*)d*E* between −0, 5 eV and the Fermi energy. While these results show trends consistent with our observations, they cannot quantitatively predict the YSR energies.

For molecular orientations ranging between 25 and 45°, the calculated LUMO energy *E*_L_ is lowest for SnPc↓ on a top site and highest for SnPc↓ on a hollow site (with energy difference of ≈25 meV). SnPc↑ molecules on top and hollow sites fall between these values. The hybridization Γ_L_ is ≈1.4 times stronger for SnPc↓ on a hollow site compared with SnPc↑ at the same site and orientation. A shift of the orbital energy toward the empty-orbital regime and stronger hybridization, as seen for SnPc↓ on hollow sites, are less favorable for sustaining magnetic states according to the phase diagram of the Anderson impurity model.^32^ This may explain why no YSR states are observed for these molecules. In contrast, SnPc↓ on top sites, which exhibit pronounced YSR states, have lower LUMO energies, weaker hybridization, and the highest calculated LUMO occupation (*n*_L_) among all configurations, making them more conducive to magnetic states. Since the above considerations are based solely on DFT calculations, experimental determination of the LUMO energy and width from molecules within islands would provide valuable support for the analysis. However, d*I*/d*V* spectra recorded over a broader bias voltage range (see Supporting Figure 4) are significantly distorted by inelastic excitations of molecular vibrations, preventing a definitive determination of the LUMO energy and hybridization.

## Conclusions

In conclusion, SnPc molecules on Pb(100) were systematically studied, revealing distinct adsorption sites, molecular orientations, and superlattices for SnPc↑ and SnPc↓ molecules. The orientations of isolated molecules were successfully reproduced using DFT, demonstrating that molecular orientation can serve as an indicator of the adsorption site. The energies of molecular σ-orbitals were significantly lowered when lone pairs on the bridging-aza nitrogen atoms pointed toward substrate Pb atoms. Conversion from SnPc↑ to SnPc↓ was achieved through electron extraction from the HOMO orbital, which is associated with a reduction of the switching barrier according to DFT calculations. In self-assembled islands, molecular orientations and adsorption sites were observed that deviate from those of isolated molecules. Notably, strong YSR states–indicative of a molecular spin–were induced by positioning SnPc↓ molecules on top sites. This behavior was rationalized based on calculated LUMO energies, providing insight into the spin-dependent properties of these systems.

## Methods

Pb(100) single crystal surfaces were prepared by repeated cycles of Ar-ion sputtering and subsequent annealing to approximately 500 K. STM-tips were cut from a Pb wire and sputtered in ultrahigh vacuum (UHV). SnPc molecules were deposited in situ from a Knudsen cell onto the Pb substrate, which was kept close to room temperature. All measurements were carried out with UHV scanning tunneling microscopes (STM) operated at *T* = 4.2 K. STM topographs were obtained using at *I* = 100 pA unless otherwise noted. Measurements of the differential conductance d*I*/d*V* were made by freezing the position of the STM tip and using standard lock-in technique with a sinusoidal modulation (frequency 831 Hz) added to *V*. To prepare artificial molecular arrangements, single molecules were laterally moved on the sample surface using *V* = 4 mV and slightly elevated currents of ≈4 and 9 nA at for SnPc↑ and SnPc↓ molecules, respectively.

Calculations for adsorbed SnPc were performed using the Vienna ab initio simulation package (VASP),^33,34^ with the Perdew–Burke–Ernzerhof (PBE) functional.^35^ For geometry optimization and self-consistent calculations, a plane-wave basis set with a kinetic energy cutoff of 400 eV was chosen. The Pb(100) surface was modeled with nine layers of Pb atoms and a vacuum region of 1.8 nm thickness. All atoms, except the bottom four Pb layers were relaxed until the residual force per atom was less than 0.2 eV/nm. van der Waals (vdW) interactions were taken into account using the DFT-D3 method.^36^ The Brillouin zone was sampled by a 3 × 3 × 1 *k*-grid.

Gas phase DFT calculations were done using Gaussian^37,38^ with the Lan2DZ basis set and the B3LYP exchange correlation functional.

## Supporting Information Available

Details on the molecular manipulation and switching, d*I*/d*V* spectra of a wider voltage range, and d*I*/d*V* maps showing the spatial distribution of YSR states in islands (PDF)
