# Supplementary material for: Adsorption-Site- and Orientation-Dependent Magnetism of a Molecular Switch on Pb(100)
Source: ACS Nano. 2025 Feb 14;19(7):7231–8. doi: 10.1021/acsnano.4c17183 (PMC11867006; doi:10.1021/acsnano.4c17183)
Supplement: Supplementary file 1 — nn4c17183_si_001.pdf [file nn4c17183_si_001.pdf]

Supporting Information to Adsorption-Site and Orientation Dependent Magnetism of a Molecular Switch on Pb(100)

Arnab Banerjee,† Niklas Ide,† Yan Lu,‡

Richard Berndt,*,† and Alexander Weismann*,†

†*Institut für Experimentelle und Angewandte Physik, Christian-Albrechts-Universität zu Kiel, 24098 Kiel, Germany*

‡*Department of Physics, Nanchang University, anchang, 330031, People's Republic of China*

E-mail: berndt@physik.uni-kiel.de; weismann@physik.uni-kiel.de

Manipulation Sequence of Single Molecules from Steps

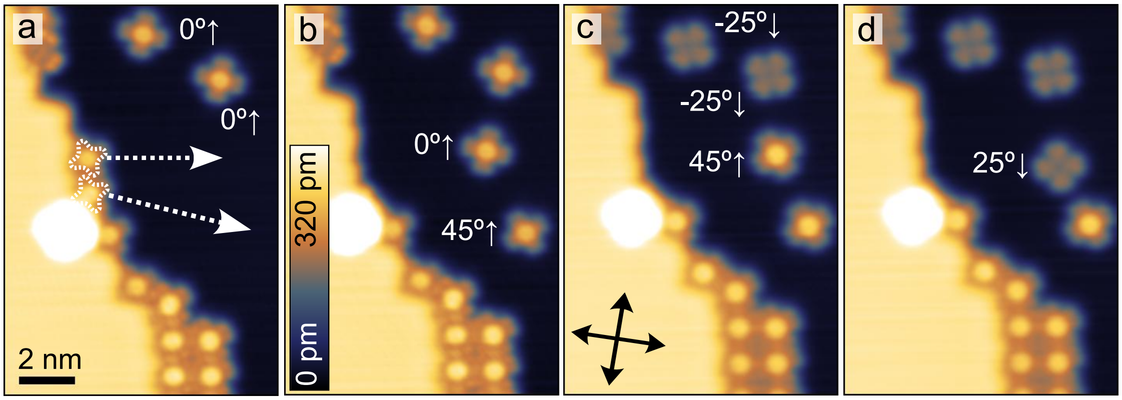

Figure S1: Sequence of topographs showing the removal of SnPc↑ molecules from an island edge (panel a) onto the pristine substrate (panel b). Subsequently, isolated SnPc↑ molecules are converted to SnPc↓ by centering the tip over a molecule and increasing the voltage. In (c), the top two molecules have been converted. In (d), a third molecule has been manipulated. The orientations of some molecules are indicated and show that the vertical translation of the Sn ion is accompanied by a rotation to the typical orientations of SnPc↓. Constant current topographs were recorded at $V = 5$ and 100 mV for (a, b) and (c, d), respectively.

Spatial Distribution of YSR-States in Islands

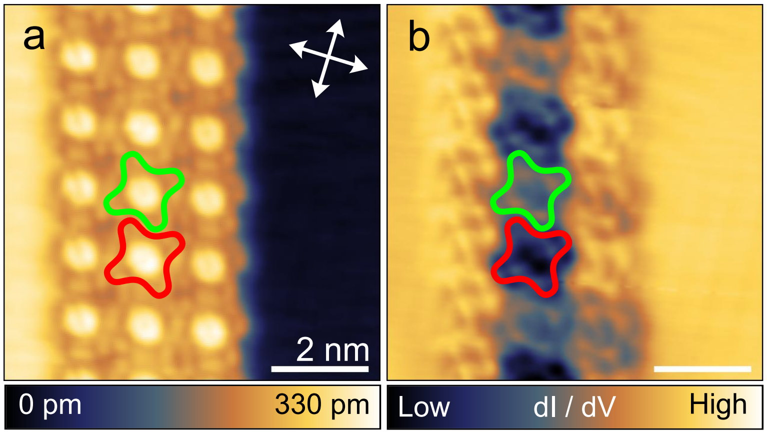

Figure S2: (a) Topograph ($V = 2.5\,\mathrm{mV}$, $I = 100\,\mathrm{pA}$) of a molecular island composed of SnPc↑ molecules. The α_1 and α_2 orientations are marked by red and green contours, respectively. (b) $\mathrm{d}I/\mathrm{d}V$-map recorded at $V = 2.5\,\mathrm{mV}$ and $I = 100\,\mathrm{pA}$. The voltage used is located slightly outside the coherence peak, where a local minimum indicates the presence of a YSR state. A significant reduction in differential conductance is observed on the α_1 molecules (red).

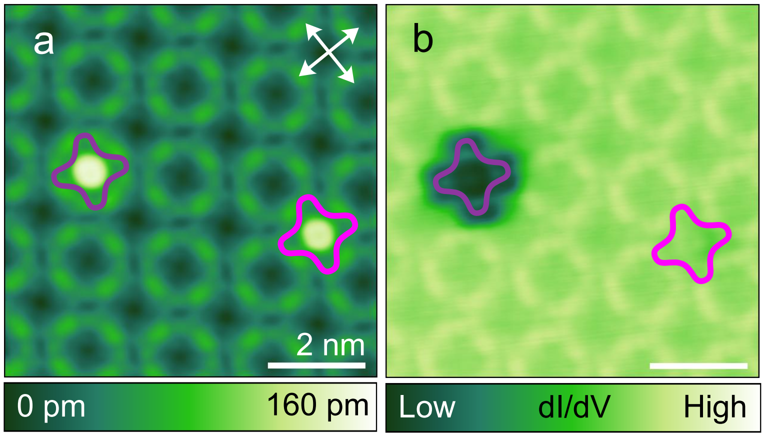

Figure S3: (a) Topograph ($V = 5\,\mathrm{mV}$, $I = 100\,\mathrm{pA}$) of a molecular island composed predominantly of SnPc↓ molecules. Two SnPc↑ are present with β_1 (top left) and β_2 (bottom right) orientations, indicated by the violet and pink contours, respectively. (b) $\mathrm{d}I/\mathrm{d}V$-map recorded at $V = 2.5\,\mathrm{mV}$ and $I = 100\,\mathrm{pA}$. At the voltage used, in the presence of a YSR state is reflected by a reduced conductance. A YSR state is observed exclusively on the β_1 molecule.

dI/dV Spectra of Molecules in Islands

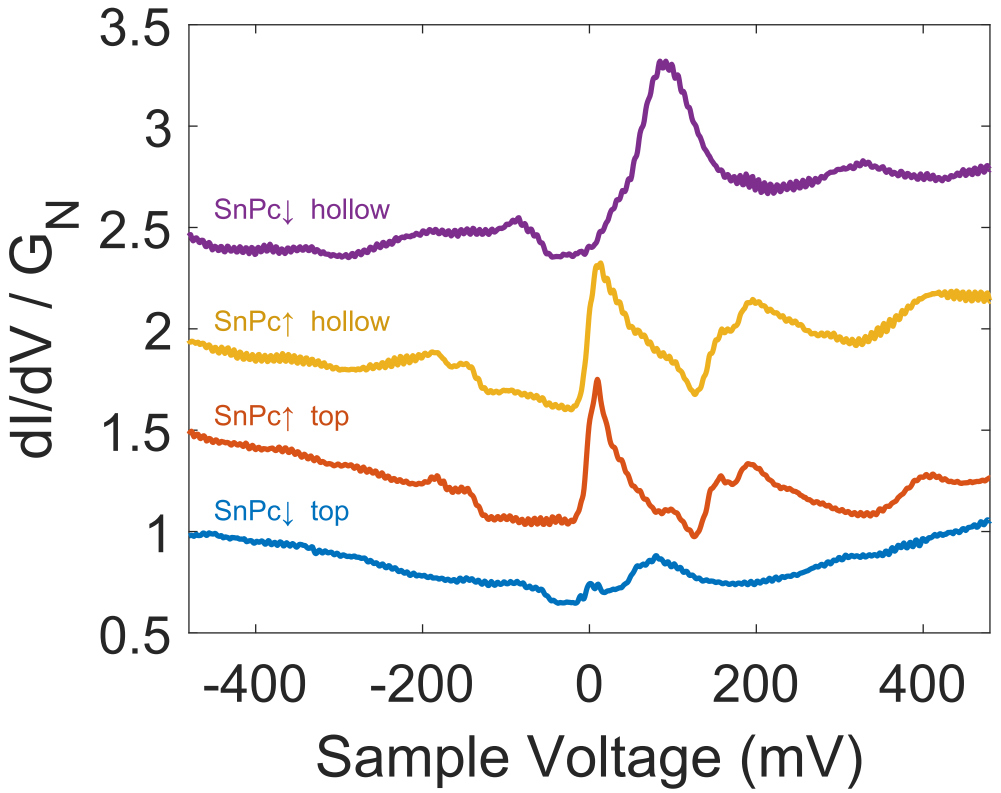

Figure S4: dI/dV spectra of molecules in islands recorded over a wide voltage range (voltage modulation $V_{mod} = 10$ and $5\,\mathrm{mV_{pp}}$ for hollow and top sites, respectively). The spectra were normalized to the same differential conductance at $-500\,\mathrm{meV}$ and vertically shifted in steps of $0.5\,G_N$ for clarity. The spectra are significantly affected by inelastic excitations of molecular vibrations, with notable steps at ± 140 and $\pm 180\,\mathrm{meV}$ for SnPc↑, $\pm 60\,\mathrm{meV}$ for SnPc↓, preventing a definitive determination of the LUMO energy.
